# Covid-19-associated pulmonary aspergillosis in mechanically ventilated patients: incidence and outcome in a French multicenter observational cohort (APICOVID)

**DOI:** 10.1186/s13613-023-01229-3

**Published:** 2024-01-29

**Authors:** Luc Desmedt, Matthieu Raymond, Aurélie Le Thuaut, Pierre Asfar, Cédric Darreau, Florian Reizine, Gwenhaël Colin, Johann Auchabie, Julien Lorber, Béatrice La Combe, Pierre Kergoat, Baptiste Hourmant, Agathe Delbove, Aurélien Frérou, Jean Morin, Pierre Yves Ergreteau, Philippe Seguin, Maëlle Martin, Jean Reignier, Jean-Baptiste Lascarrou, Emmanuel Canet

**Affiliations:** 1 Service de Médecine Intensive Réanimation, CHU Nantes, Nantes Université, 30 Bd. Jean Monnet, 44000 Nantes, France; 2 Direction de la recherche, Plateforme de Méthodologie et Biostatistique, CHU de Nantes, Nantes, France; 3 Service de Médecine Intensive Réanimation, CHU d’Angers, Angers, France; 4 Service de Réanimation polyvalente, CH du Mans, Le Mans, France; 5 Service de Médecine Intensive Réanimation, CHU de Rennes, Rennes, France; 6 Service de Réanimation polyvalente, CHD de La Roche sur Yon, La Roche-sur-Yon, France; 7 Service de Réanimation polyvalente, CH de Cholet, Cholet, France; 8 Service de Réanimation polyvalente, CH de Saint Nazaire, Saint-Nazaire, France; 9 Service de Réanimation Polyvalente, Groupe Hospitalier Bretagne Sud, Lorient, France; 10 Service de Réanimation polyvalente, Cornouille General Hospital, Quimper, France; 11 Service de Médecine Intensive Réanimation, CHU de Brest, Brest, France; 12 Service de Réanimation polyvalente, Centre Hospitalier Bretagne Atlantique, Vannes, France; 13 Service de Réanimation polyvalente, CH de Saint Malo, Saint-Malo, France; 14 Unité de soins intensifs de Pneumologie, CHU de Nantes, Nantes, France; 15 Service de Réanimation polyvalente, CH de Morlaix, Morlaix, France; 16 Service de Réanimation chirurgicale, CHU de Rennes, Rennes, France; 17 Service de Médecine Intensive Réanimation, Movement-Interactions-Performance, MIP, UR 4334, CHU Nantes, Nantes Université, 44000 Nantes, France

**Keywords:** Invasive pulmonary aspergillosis, COVID‑19, Mechanical ventilation, Intensive care unit

## Abstract

**Background:**

Recent studies identified coronavirus disease 2019 (COVID-19) as a risk factor for invasive pulmonary aspergillosis (IPA) but produced conflicting data on IPA incidence and impact on patient outcomes. We aimed to determine the incidence and outcomes of COVID-19-associated pulmonary aspergillosis (CAPA) in mechanically ventilated patients.

**Methods:**

We performed a multicenter retrospective observational cohort study in consecutive adults admitted to 15 French intensive care units (ICUs) in 2020 for COVID-19 requiring mechanical ventilation. CAPA was diagnosed and graded according to 2020 ECMM/ISHAM consensus criteria. The primary objective was to determine the incidence of proven/probable CAPA, and the secondary objectives were to identify risk factors for proven/probable CAPA and to assess associations between proven/probable CAPA and patient outcomes.

**Results:**

The 708 included patients (522 [73.7%] men) had a mean age of 65.2 ± 10.8 years, a median mechanical ventilation duration of 15.0 [8.0–27.0] days, and a day-90 mortality rate of 28.5%. Underlying immunosuppression was present in 113 (16.0%) patients. Corticosteroids were used in 348 (63.1%) patients. Criteria for probable CAPA were met by 18 (2.5%) patients; no patient had histologically proven CAPA. Older age was the only factor significantly associated with probable CAPA (hazard ratio [HR], 1.04; 95% CI 1.00–1.09; *P* = 0.04). Probable CAPA was associated with significantly higher day-90 mortality (HR, 2.07; 95% CI 1.32–3.25; *P* = 0.001) but not with longer mechanical ventilation or ICU length of stay.

**Conclusion:**

Probable CAPA is a rare but serious complication of severe COVID-19 requiring mechanical ventilation and is associated with higher day-90 mortality.

**Graphical Abstract:**

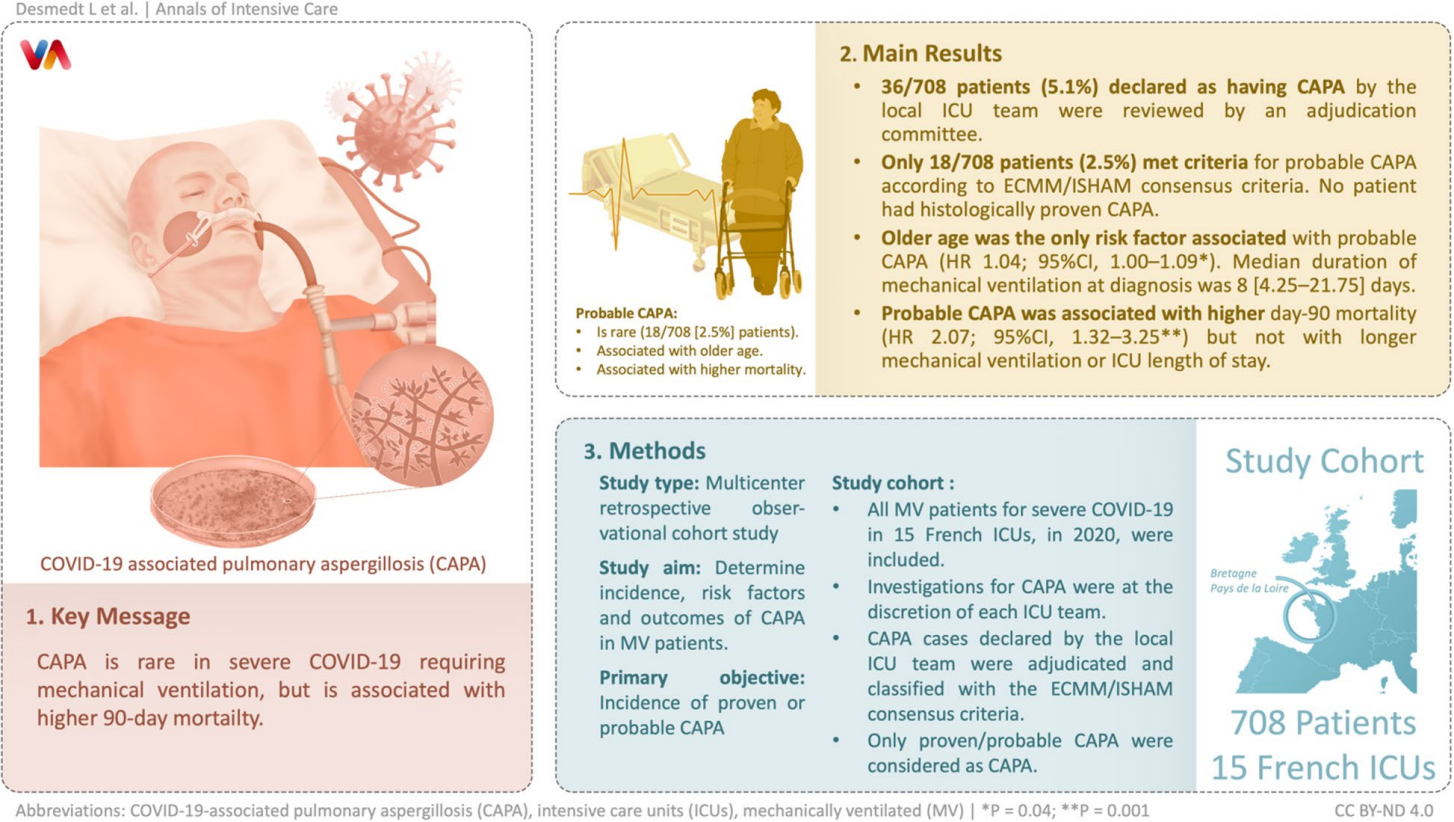

**Supplementary Information:**

The online version contains supplementary material available at 10.1186/s13613-023-01229-3.

## Background

Invasive pulmonary aspergillosis (IPA) is increasingly recognized as a complication of severe COVID-19 in patients who lack host factors traditionally associated with invasive fungal disease [1]. Virus-induced damage to the airway epithelium combined with immune response impairments due to the pro-inflammatory cytokine storm and/or corticosteroid therapy may explain the increased risk of IPA in patients with COVID-19 critical illness [2].

The diagnosis of COVID-19-associated pulmonary aspergillosis (CAPA) is extremely challenging since the clinical and radiographic manifestations closely resemble those produced by bacterial superinfection during COVID-19 [3]. Consequently, current case definitions rely chiefly on the results of mycological tests on bronchoalveolar lavage (BAL) fluid [4, 5]. Given the absence of histopathology-controlled studies, a major drawback of these case definitions is their unknown ability to distinguish between colonization and infection.

Across studies, the incidence of CAPA varied widely, from 2.5 to 28%, depending on factors such as the screening policy, COVID-19 severity, and definitions used [6–11]. Interestingly, a 2022 systematic review suggests that reported CAPA prevalences may be overestimated due to the use of non-standard case definitions: when CAPA cases were reviewed by an independent committee, the value dropped from 10 to 4% [12]. Finally, the impact of CAPA on patient outcomes varied across large multicenter cohorts, and antifungal treatment was not associated with improved survival [6, 7, 11].

The main objective of this study was to assess the incidence of CAPA in a large multicenter cohort of patients admitted to the intensive care unit (ICU) for severe COVID-19 requiring endotracheal mechanical ventilation (MV). The secondary objectives were to identify risk factors for CAPA and to assess associations linking CAPA to patient outcomes. We hypothesized that CAPA was a rare complication of severe COVID-19 and was associated with worse outcomes.

## Methods

This study was approved by the ethics committee of the French Intensive Care Society (CE SRLF 21-07) on February 11, 2021. In accordance with French law on retrospective studies of anonymized healthcare data, informed consent was not required. This report complies with STROBE guidelines [13].

### Study design and population

We performed a multicenter retrospective observational cohort study in consecutive patients admitted to any of 15 French ICUs between February 1 and December 31, 2020 (Additional file 1: Table S1). Inclusion criteria were age ≥ 18 years, positive SARS-CoV-2 polymerase-chain-reaction (PCR) test on a nasopharyngeal swab or respiratory sample, manifestations of lower respiratory tract infection (fever, dyspnea, and radiographic lung infiltrates), and MV. No patients meeting the inclusion criteria were excluded.

### Data collection and case definition

For each patient, a local investigator at each ICU entered data from the ICU records into a standardized web-based electronic case-report form (Castor® Electronic Data Capture System, Amsterdam, The Netherlands). For each patient with CAPA, the clinical, radiological, and microbiological data were collected.

Investigations for CAPA were at the discretion of each ICU team, given the uncertainty about the optimal diagnostic workup [5, 6]. Investigations could be triggered by unexplained clinical/radiological deterioration and/or by the results of routine screening. In centers that practiced routine screening, mycological culture of a tracheal aspirate was performed once a week. All mycological tools for diagnosing CAPA (*Aspergillus* PCR, galactomannan assay, microscopic smear examination, and mycological culture) were available to all centers, either locally or by sending samples to one of the four university hospitals involved in the study. All but two centers could obtain smear examinations and mycological cultures at their local microbiology laboratory. *Aspergillus* PCR and galactomannan assays were performed at the four participating university hospitals.

The data for each patient recorded as having CAPA by the local investigator were reviewed by an adjudication committee of three independent experts, who applied the 2020 ECMM/ISHAM consensus criteria for CAPA diagnosis and classification as possible, probable, or proven [5] (Additional file 1: Table S2). For this study, CAPA was defined as disease meeting criteria for either probable or proven CAPA. Patients with possible CAPA were not eligible for study inclusion. No center used antifungal prophylaxis to prevent CAPA.

Ventilator-associated pneumonia (VAP) was defined as concomitant with CAPA when diagnosed within 48 h before or after CAPA (Additional file 1: Appendix S1).

Patients were classified as immunocompromised if they had any of the following: solid-organ transplantation, human immunodeficiency virus infection, hematopoietic stem-cell transplantation, hematological malignancy, solid malignancy (new diagnosis or current progression or in remission for less than 5 years), corticosteroid or other immunosuppressant therapy for longer than 30 days before COVID-19 onset, or known primary immunodeficiency.

### Objectives

The primary study objective was to assess the incidence of disease meeting ECMM/ISHAM consensus criteria for probable or proven CAPA (designated “CAPA” hereafter) [5]. The secondary objectives were to identify risk factors for CAPA and to assess potential associations linking CAPA to MV duration, ICU length of stay, and day-90 mortality.

### Statistical analysis

In the overall population and in the groups with and without probable/proven CAPA, quantitative variables were described as mean ± SD if normally distributed and as median [interquartile range] otherwise. Categorical variables were described as *n* (%). Comparisons of quantitative variables were with Student’s *t* test or the Mann–Whitney test, depending on normality; for categorical variables, we used the chi-square test or Fisher’s exact test, as appropriate. Baseline patient features in the groups with vs. without proven/probable CAPA were compared using Student’s *t* test or the Wilcoxon Mann–Whitney test if quantitative and Pearson’s chi-square or Fisher’s test if categorical.

We performed exploratory analyses to look for associations linking CAPA to MV duration, ICU length of stay, and day-90 mortality. Overall day-90 survival was analyzed using time-dependent Cox proportional hazards models. For MV and ICU stay durations, we built a Fine-and-Gray model with death as a competing risk (additional file, methods section). CAPA occurrence was fitted as a time-dependent variable. Confounding factors considered for the multivariate model were age, Sequential Organ Failure Assessment (SOFA) score at ICU admission, Charlson’s Comorbidity Index, time from symptom onset to ICU admission, and immunocompromised status. Missing data were ignored.

The statistical analyses were done using SAS 9.4 (SAS institute, Cary, NC). *P* values < 0.05 were taken to indicate significant differences.

## Results

### Patient characteristics at ICU admission

Table 1 shows that the baseline features of the 708 included patients were typical of severe COVID-19, with a predominance of elderly males and a high prevalence of comorbidities. Two-thirds of patients received corticosteroid therapy, whereas only 4 (0.57%) patients were given an IL-6 antagonist. The only baseline variable that differed significantly between the groups with vs. without CAPA by univariate analysis was age, which was older in the CAPA group.

**Table 1:** Baseline features of the study patients

Variable (*n* of patients with available data)	All patients (*n* = 708)	Probable^a^ CAPA (*n* = 18)	No CAPA^a^ (*n* = 690)	Univariate HR (95% CI)	*P* value
Demographics, mean ± SD or *n* (%)					
Age (*n* = 708)	65 ± 11	70 ± 7	65 ± 11	1.04 (1.00–1.09)	0.04
Male (*n* = 708)	522 (73.7)	15 (83.3)	507 (73.5)	1.56 (0.45–5.38)	0.48
BMI (kg/m^2^) (*n* = 704)	30 ± 6	28 ± 5	30 ± 6	0.95 (0.87–1.03)	0.20
Severity scores, mean ± SD					
SAPS II (*n* = 642)	39 ± 13	39 ± 13	39 ± 13	0.99 (0.96–1.03)	0.69
SOFA score (*n* = 626)	5 ± 3	4 ± 2	5 ± 3	0.88 (0.76–1.02)	0.09
Comorbidities, mean ± SD or *n* (%)					
Charlson’s Comorbidity Index (*n* = 703)	4 ± 3	5 ± 3	4 ± 3	1.07 (0.92–1.24)	0.41
Diabetes (*n* = 708)	222 (31.4)	3 (16.7)	219 (31.7)	0.42 (0.12–1.40)	0.16
Hypertension (*n* = 708)	387 (54.7)	10 (55.6)	377 (54.6)	0.99 (0.39–2.51)	0.98
Chronic respiratory failure (*n* = 708)	138 (19.5)	5 (27.8)	133 (19.3)	1.49 (0.53–4.17)	0.45
Chronic heart failure (*n* = 708)	111 (15.7)	3 (16.7)	108 (15.7)	1.03 (0.30–3.50)	0.96
Chronic kidney failure (*n* = 708)	68 (9.6)	1 (5.6)	67 (9.7)	0.48 (0.07–3.49)	0.47
Cirrhosis (*n* = 708)	40 (5.7)	2 (11.1)	38 (5.5)	1.91 (0.43–8.51)	0.40
Any immunosuppression (*n* = 708)	113 (16)	4 (22.2)	109 (15.8)	1.32 (0.42–4.09)	0.63
Solid organ transplantation (*n* = 708)	18 (2.5)	0 (0.0)	18 (2.6)	NA	NA
Active hematological or solid malignancy (*n* = 708)	58 (8.2)	3 (16.7)	55 (8)	1.81 (0.50–6.50)	0.36
HIV (*n* = 708)	7 (1)	1 (5.6)	6 (0.9)	7.51 (0.88–64.0)	0.07
Immunosuppressive drugs (*n* = 708)	35 (4.9)	1 (5.6)	34 (4.9)	1.15 (0.16–8.56)	0.89
Treatments for COVID-19, *n* (%)					
Corticosteroids (*n* = 707)	446 (63.1)	12 (66.7)	434 (63)	1.08 (0.39–3.01)	0.88
IL6 antagonist (*n* = 699)	4 (0.6)	0 (0.0)	4 (0.6)	NA	NA
Hydroxychloroquine (*n* = 708)	92 (13)	2 (11.1)	90 (13)	0.88 (0.20–3.81)	0.87
Lopinavir/rotinavir (*n* = 708)	88 (12.4)	4 (22.2)	84 (12.2)	1.79 (0.62–5.20)	0.28
Remdesivir (*n* = 708)	26 (3.7)	0 (0.0)	26 (3.8)	NA	NA

*CAPA* COVID-19-associated pulmonary aspergillosis, *HR* hazards ratio, *BMI* Body Mass Index, *SAPS II* Simplified Acute Physiology Score version II, *SOFA* Sequential Organ Failure Assessment, *95% CI* 95% confidence interval, *HIV* human immunodeficiency virus, *NA* not available

^a^According to 2020 ECMM/ISHAM consensus criteria

### Incidence of CAPA and characteristics of patients with CAPA

**Fig. 1:**
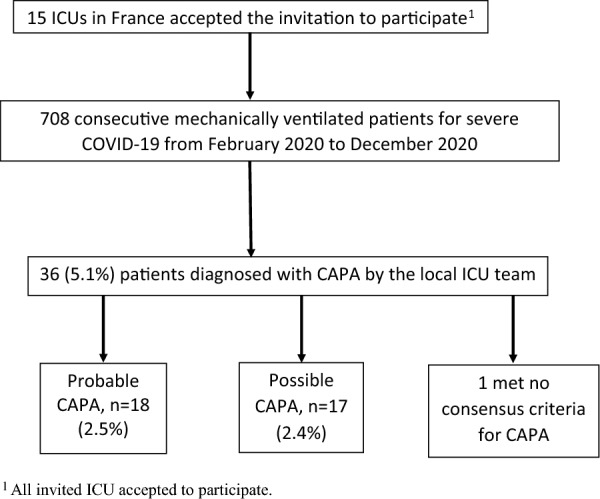
Patient flowchart. *COVID-19* coronavirus disease 2019, *MV* endotracheal mechanical ventilation, *ICU* intensive care unit, *CAPA* COVID-19-associated pulmonary aspergillosis. Proven, probable, and possible CAPA were defined according to the 2020 ECMM/ISHAM consensus criteria

Figure 1 is the flowchart. Of the 708 patients, 36 (5.1%) had suspected CAPA according to local ICU teams, including 18 (18/708, 2.5%) who had probable CAPA according to the adjudication committee. The incidence of probable CAPA ranged across centers from 8.6% (6/70) to 0% (0/111) (Additional file 1: Table S1). The incidence of CAPA was higher in the two centers that performed routine screening compared to the other 13 centers (1.7% [9/541] vs 5.4% [9/167], respectively; *P* = 0.03). The incidence of CAPA was not significantly higher in the six centers that had all mycological tools locally (3.1% [14/450], compared to 1.6% [4/258] in the nine centers that sent samples university hospitals; *P* = 0.3). A postmortem lung biopsy was performed in 19 patients (2 with and 17 without CAPA) and was consistently negative: thus, no patient had proven CAPA. The 17 patients who met criteria for possible CAPA were not considered for this study (Additional file 1: Tables S3 and S4).

Table 2 displays the main manifestations in the patients with CAPA and shows the numbers of patients who underwent each investigation. Median MV duration at diagnosis was 8 [4.3–21.8] days. The clinical and radiological abnormalities were not specific. A third of patients had concomitant bacterial VAP. A BAL smear or culture positive for *Aspergillus* was the most common finding leading to the diagnosis (13/18, 76%). Serum galactomannan was positive in only 3/13 (23%) patients. Only 5/18 (28%) patients had two or more positive mycological criteria. A postmortem lung biopsy was performed in 2 patients, neither of whom had histopathological evidence of invasive aspergillosis.

**Table 2:** Main manifestations in the 18 patients with probable COVID-19-associated pulmonary aspergillosis

Time from ICU admission to diagnosis, median [IQR]	9.5 [7.00–21.75]
Time from intubation to diagnosis, median [IQR]	8 [4.25–21.75]
Clinical presentation (18 patients), *n* (%)	
Hemoptysis	1 (5)
Refractory fever	9 (50)
Respiratory function deterioration	18 (100)
Concomitant VAP^a^	6 (33)
Chest computed tomography scan (18 patients), *n* (%)	
Interstitial infiltrate	18 (100)
Cavity	1 (5)
Nodules	3 (17)
Consolidation	16 (89)
Macroscopic appearance by fiberoptic bronchoscopy (3 patients), *n* (%)	
Normal	2 (66)
Nodules	1 (33)
Serum galactomannan index (13 patients), *n* (%)	
Galactomannan index > 0.5	3 (23)
Fiberoptic bronchoscopy with bronchoalveolar lavage (17 patients), *n* (%)	
Galactomannan index ≥ 1	4/13 (31)
Positive *Aspergillus* PCR	3/3 (100)
Direct microscopy and/or mycological culture positive for *Aspergillus*	13/17 (76)
Multiple positive mycological criteria^b^ (≥ 2), *n* (%)	5/18 (28)

*VAP* ventilator-associated pneumonia, *PCR* polymerase chain reaction

^a^Defined as VAP defined within 48 h before or after CAPA

^b^According to the mycological criteria defined by the ECMM/ISHAM consensus (Additional file 1: Table S2)

Antifungal treatment was given to 17/18 (94%) patients. Voriconazole was the first-line drug in 15 patients, and 5 patients received more than one antifungal. Median antifungal treatment duration was 19.5 [12.5–30.5] days (Additional file 1: Table S5).

### Outcomes in patients with probable COVID-19-associated pulmonary aspergillosis

**Fig. 2:**
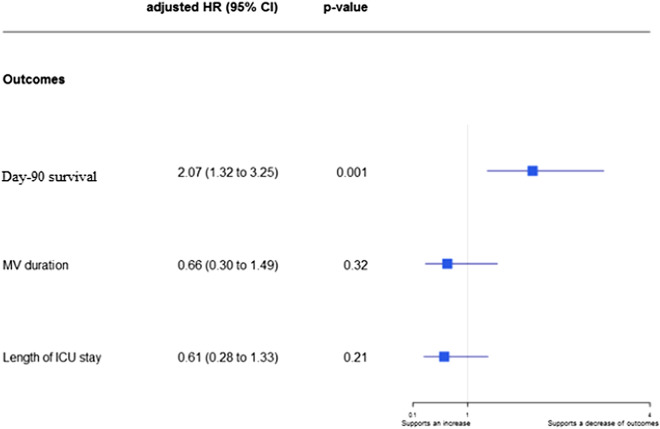
Association of probable CAPA with day-90 outcomes. Survival was analyzed using time-dependent Cox proportional hazards models. Mechanical ventilation duration and length of ICU stay were analyzed using a Fine and Gray model. IPA occurrence was fitted as a time-dependent variable. Adjusted HRs were calculated by including age, SOFA score, Charlson’s Comorbidity Index, time from symptom onset to ICU admission, and immunocompromised status as pre-specified covariates. HR > 1 indicates a decrease in survival (i.e., increased mortality), a shorter MV duration (i.e., an increased likelihood of survival with extubation), or a shorter ICU stay (i.e., an increased likelihood of discharge alive). HR < 1 indicates an increase in survival (i.e., decreased mortality), longer MV duration (i.e., a decreased likelihood of survival with extubation), or a longer ICU stay (i.e., a decreased likelihood of discharge alive). *CAPA* COVID-19-associated pulmonary aspergillosis, *IPA* invasive pulmonary aspergillosis, *95% CI* 95% confidence interval, *HR* hazard ratio, *ICU* intensive care unit, *MV* endotracheal mechanical ventilation, *SOFA* Sequential Organ Failure Assessment

MV duration, ICU length of stay, and day-90 mortality were higher in the group with vs. without CAPA (Table 3). By univariate analysis with CAPA handled as a time-dependent event, day-90 mortality was significantly higher in the CAPA group (hazard ratio [HR], 2.56; 95% confidence interval [95% CI], 1.59–4.12; *P* = 0.001). CAPA was not significantly associated with MV duration (HR, 0.53; 95% CI 0.24–1.18; *P* = 0.12) or ICU length of stay (HR, 0.53; 95% CI 0.24–1.18; *P* = 0.065). Multivariate analyses produced similar findings (Fig. 2; Additional file 1: Tables S6, S7, and S8).

**Table 3:** Outcomes in the groups with vs. without COVID-19-associated pulmonary aspergillosis according to 2020 ECMM/ISHAM consensus criteria

	All patients (*n* = 708)	Probable CAPA (*n* = 18)	No CAPA (*n* = 690)
Day-90 mortality, *n* (%)	202 (28.5)	10 (55.6)	192 (27.8)
MV duration, days, median [IQR]	15.0 [8.00–27.00]	27.0 [18.00–34.00]	15.0 [8.00–26.00]
ICU length of stay, days, median [IQR]	19.0 [11.00–31.00]	26.5 [19.00–54.00]	19.0 [11.00–31.00]

Outcomes were available for all patients included in the study

*CAPA* COVID-19-associated pulmonary aspergillosis, *95% CI* 95% confidence interval, *MV* endotracheal mechanical ventilation, *ICU* intensive care unit

## Discussion

In this large retrospective multicenter cohort of patients who required ICU admission and MV for severe COVID-19, CAPA was uncommon. We did not consider possible disease, and no patients had proven disease; 18 (2.5%) had probable CAPA. The only risk factor was older age. CAPA was associated with higher day-90 mortality but not with longer time on MV or in the ICU.

The low incidence of CAPA in our cohort is in accordance with previous findings from large multicenter studies [8, 10, 14] and an autopsy study [15]. Higher incidences have been reported, chiefly when routine screening was performed [2, 10, 11]. Only two of the 15 ICUs applied routine screening. A drawback of routine screening is the risk of overdiagnosis due to difficulties in distinguishing colonization from infection. A third of our patients had concomitant bacterial VAP. This common complication of severe COVID-19 occurred in nearly half the patients in a previous study [16]. Clinical deterioration in patients with severe COVID-19 may indicate VAP, but CAPA should be suspected if no improvement occurs with adequate antibiotic treatment. Otherwise, a combination of clinical and radiological features due to VAP and of mycological findings due to colonization might lead to an erroneous diagnosis of CAPA. To our knowledge, data on concomitant VAP were not collected in studies of routine screening [6, 17, 18].

In our cohort, CAPA occurred chiefly in older patients after 4 days of MV. As previously reported, serum galactomannan was rarely positive and the diagnosis relied chiefly on BAL results [17, 18]. Thus, when CAPA is suspected, BAL is the specimen of choice [5]. The absence of associations linking CAPA to EORTC/MSG host factors, corticosteroid therapy, and COPD conflicts with earlier data [6, 7, 14, 19, 20]. This discrepancy may be due to the small number of patients with CAPA, which resulted in low statistical power. Larger sample sizes in earlier studies were often related to the inclusion of possible cases of CAPA, which were not considered in our study. The number of patients given IL-6 antagonist therapy (tocilizumab) to treat COVID-19 was too small (*n* = 4) to allow a statistical analysis of this factor.

By univariate and multivariate analyses, CAPA was significantly associated with higher day-90 mortality but not with longer ICU stay or MV duration. The absence of associations with ICU stay and MV durations may be related to the small sample size. However, in a recent meta-analysis, neither the ICU stay nor the MV duration was significantly longer in patients with probable CAPA than in patients without CAPA [19]. We were unable to assess potential associations between antifungal therapy and CAPA outcomes since all patients but one received antifungal therapy. To date, no study has demonstrated that antifungal treatment improves survival, even when CAPA is diagnosed early by routine screening [6, 11, 17].

Our study has several limitations that may have resulted in underestimation of the incidence of CAPA. First, the design was retrospective. Second, all patients were included early in the COVID-19 pandemic, when concern existed about the risk of viral spread by aerosolization during bronchoscopy. Given the major role for BAL in diagnosing CAPA, the resulting reluctance to perform BAL may have led to CAPA cases being missed. Third, CAPA was not as well recognized during the study period as it is now, and the index of suspicion may, therefore, have been insufficient. Since our work, international guidelines and numerous studies have been published [5, 6]. Fourth, mycological tools were not standardized across the participating ICUs, and the full spectrum of investigations for aspergillosis (notably *Aspergillus* PCR and galactomannan assay) may not have been performed in all patients with suspected CAPA. Neither were the criteria for suspecting CAPA standardized: the index of suspicion may have been higher in some participating ICUs than in others. That our results were consistent across centers suggests a limited impact of these weaknesses: importantly, the incidence of CAPA was low even in the centers practicing routine screening and in those with local access to all mycological tests. Interestingly, two patients who met criteria for probable CAPA had postmortem examinations with no evidence of IPA, highlighting the possible overestimation of the disease related to sub-optimal case definitions. These weaknesses reflect the need for further work aimed at developing tests and diagnostic algorithms for CAPA. Fifth, our results may not be applicable to the current COVID-19 endemicity, variants, and treatments. Notably, corticosteroids were given to only about two-thirds of patients and IL-6 antagonist therapy was very rarely used. Previous studies demonstrated associations linking these two treatments to the occurrence of CAPA [6, 7]. Moreover, all participating ICUs were in western France, and our findings may not apply to other parts of the world [21]. Finally, the association of CAPA with day-90 mortality demonstrated in our study may have been biased by the overlap between manifestations indicating greater respiratory disease severity (possibly due to COVID-19 or CAPA or VAP) and, therefore, a higher risk of death, and manifestations prompting tests for CAPA.

Strengths of our study include the large number of centers and patients receiving MV for COVID-19, the use of ECCM/ISHAM criteria to define probable and proven CAPA, and the review of each suspected CAPA case by an adjudication committee composed of three independent experts.

In summary, the incidence of CAPA was considerably lower than reported by others. This result may be ascribable to several limitations of our study potentially associated with underestimation of the incidence of CAPA, notably the recruitment confined to the first two COVID-19 waves and to western France. Our findings may not apply to other geographic areas or to COVID-19 cases occurring now, given the changes over time in the management of COVID-19 and virulence of the SARS-CoV-2 virus. Nevertheless, given the low incidence and absence of proven effects of curative antifungal therapy, both routine screening and prophylactic therapy in patients on MV for severe COVID-19 would seem unreasonable. We suggest that testing for CAPA should be performed only in patients with unexplained respiratory-function deterioration, notably after a week of MV.

## Conclusion

CAPA is a rare but serious complication of severe COVID-19 requiring ICU admission and MV. In our cohort, CAPA was significantly associated with higher day-90 mortality. Our data support neither routine screening nor prophylactic treatment for aspergillosis. Studies are needed to identify the manifestations that should trigger investigations for aspergillosis, develop improved diagnostic strategies, and assess the impact on outcomes of antifungal treatment in patients with probable or proven CAPA.

### Supplementary Information

**Additional file 1: Appendix S1**. Additional methods. **Table S1.** Distribution of included patients according to participating ICU and CAPA status. **Table S2.** ECMM/ISHAM consensus criteria. **Table S3.** Baseline features at ICU admission of patients who met 2020 ECMM/ISHAM consensus criteria for possible CAPA compared to patients who met no criteria for CAPA. **Table S4.** Possible CAPA defined by 2020 ECMM/ISHAM consensus criteria: association with outcomes. **Table S5.** Additional information about antifungal treatment in CAPA patients. **Table S6.** Association between CAPA and MV duration by multivariate model. **Table S7.** Association between CAPA and ICU length of stay by multivariate model. **Table S8.** Association between CAPA and day-90 mortality by multivariate model.

## Data Availability

The datasets used and/or analyzed during the current study are available from the corresponding author on reasonable request.

## Abbreviations

95%CI: 95% Confidence interval
aHR: Adjusted hazard ratio
BAL: Bronchoalveolar lavage
CAPA: COVID‑19‑associated invasive pulmonary aspergillosis
COPD: Chronic obstructive pulmonary disease
COVID: Coronavirus disease
HR: Hazard ratio
ICU: Intensive care unit
IPA: Invasive pulmonary aspergillosis
MV: Endotracheal mechanical ventilation
PCR: Polymerase chain reaction
SARS‑CoV‑2: Severe acute respiratory syndrome coronavirus 2
VAP: Ventilator-associated pneumonia
