# Supplementary material for: Covid-19-associated pulmonary aspergillosis in mechanically ventilated patients: incidence and outcome in a French multicenter observational cohort (APICOVID)
Source: Ann Intensive Care. 2024 Jan 29;14:17. doi: 10.1186/s13613-023-01229-3 (PMC10825096; doi:10.1186/s13613-023-01229-3)
Supplement: Supplementary file 1 — Additional file 1: Appendix S1. Additional methods. Table S1. Distribution of included patients according to participating ICU and CAPA status. Table S2. ECMM/ISHAM consensus criteria. Table S3. Baseline features at ICU admission of patients who met 2020 ECMM/ISHAM consensus criteria for possible CAPA compared to patients who met no criteria for CAPA. Table S4. Possible CAPA defined by 2020 ECMM/ISHAM consensus criteria: association with outcomes. Table S5. Additional information about antifungal treatment in CAPA patients. Table S6. Association between CAPA and MV duration by multivariate model. Table S7. Association between CAPA and ICU length of stay by multivariate model. Table S8. Association between CAPA and day-90 mortality by multivariate model. [file 13613_2023_1229_MOESM1_ESM.docx]

**ADDITIONAL FILE**

**Covid-19-Associated Pulmonary Aspergillosis in Mechanically Ventilated Patients: Incidence and Outcome in a French Multicenter Observational Cohort (APICOVID)**

Luc Desmedt^1^, Matthieu Raymond^1^, Aurélie Le Thuaut^2^, Pierre Asfar^3^, Cédric Darreau^4^, Florian Reizine^5^, Gwenhaël Colin^6^, Johann Auchabie^7^, Julien Lorber^8^, Béatrice La Combe^9^, Pierre Kergoat^10^, Baptiste Hourmant^11^, Agathe Delbove^12^, Aurélien Frérou^13^, Jean Morin^14^, Pierre Yves Ergreteau^15^, Philippe Seguin^16^, Maëlle Martin^1^, Jean Reignier^17^, Jean-Baptiste Lascarrou^1^, Emmanuel Canet^1^

^1^ Nantes Université, CHU Nantes,​ Médecine Intensive Réanimation, F-44000 Nantes, France

^2^ Direction de la recherche, Plateforme de Méthodologie et Biostatistique, CHU de Nantes, France

^3^ Service de Médecine Intensive Réanimation, CHU d’Angers, France

^4^ Service de réanimation polyvalente, CH du Mans, France

^5^ Service de Médecine Intensive Réanimation, CHU de Rennes, France

^6^ Service de Médecine Intensive Réanimation, CHD de La Roche sur Yon, France

^7^ Service de réanimation polyvalente, CH de Cholet, France

^8^ Service de réanimation polyvalente, CH de Saint Nazaire, France

^9^ Service de Réanimation Polyvalente, Groupe Hospitalier Bretagne Sud, Lorient, France

^10^ Service de réanimation polyvalente, Cornouille General Hospital, Quimper, France

^11^ Service de Médecine Intensive Réanimation, Brest University Hospital, Brest, France

^12^ Service de réanimation polyvalente, Centre Hospitalier Bretagne Atlantique, Vannes, France ^13^ Service de réanimation polyvalente, CH de Saint Malo, France

^14^ Unité de soins intensifs de Pneumologie, CHU de Nantes, France

^15^ Service de réanimation polyvalente, CH de Morlaix, France

^16^ Service de réanimation chirurgicale, CHU de Rennes, France

^17^ Nantes Université, CHU Nantes,​ Médecine Intensive Réanimation, Movement - Interactions - Performance, MIP, UR 4334, F-44000 Nantes, France

**Running title**: COVID-19 and Aspergillosis: Incidence and Outcomes

# Corresponding author: Luc DESMEDT, Medical Intensive Care Unit, CHU de Nantes, 30 Bd. Jean Monnet, 44000 Nantes, FRANCE

E-mail: [luc.desmedt@chu-nantes.fr](mailto:luc.desmedt@chu-nantes.fr)

# Alternative corresponding author: Emmanuel CANET, Medical Intensive Care Unit, CHU de Nantes, 30 Bd. Jean Monnet, 44000 Nantes, FRANCE

E-mail: [emmanuel.canet@chu-nantes.fr](mailto:emmanuel.canet@chu-nantes.fr)

**CONTENTS**

**e-Appendix 1. Supplementary methods**………………………………………………… p 4

**Data collection and case definition: Lung biopsy**…………………………….... p 4

**Data collection and case definition: Ventilator-associated pneumonia**………. p 4

**Statistical analysis: Fine-and-Gray models**…………………………………..... p 5

**Distribution of included patients according to participating ICU and CAPA status: Table S1**…………………………………………………………………………………… p 6

**ECMM/ISHAM consensus criteria: Table S2**………………………………………….. p 8

**Additional information about possible CAPA: baseline characteristics and**

**outcomes**…………………………………………………………………………………. p 9

**Table S3**: Baseline features at ICU admission of patients who met 2020 ECMM/ISHAM consensus criteria for possible CAPA compared to patients who met no criteria for CAPA. ……………………………………………………………………….. p 9

**Table S4**. Possible CAPA defined by 2020 ECMM/ISHAM consensus criteria: association with outcomes…….………………………………………………………….. p 10

**Additional information about antifungal treatment in CAPA patients: Table S5...**… p 11

**Association between CAPA and MV duration, ICU length of stay, day-90 mortality. Results of multivariate models.** ………………………………………………………… p 12

**Table S6.** Duration of mechanical ventilation.………………………………….... p 12

**Table S7**. ICU length of stay………………...…………………………………… p 12

**Table S8.** Day-90 mortality. ………………...………………………………….... p 13

**e-Appendix 1. Supplementary methods**

**Data collection and case definition: Post mortem lung biopsy**

During the study period, some centres (5/15, 33%) were involved in an observational multicentre study investigating the pathological patterns of COVID-19 patients with post-mortem lung biopsies. Two transcutaneous lung biopsies per patient were performed immediately after the death: one was anterior in the second intercostal space, the second was posterior in the fifth intercostal space on the axillary line. The pulmonary side was chosen by the intensivist in charge. All specimens were fixed with 4% neutral analyzed by a group of pathologists blinded to clinical information.

**Data collection and case definition: Ventilator-associated pneumonia**

VAP was defined as hospital-acquired pneumonia diagnosed after at least 48 h of invasive mechanical ventilation or within 48 hours after extubation. The diagnosis of VAP was established by the intensivist in charge of the patient using criteria recommended by the European Centre for Disease Prevention and Control : new radiological lung infiltrates combined with at least one systemic sign (temperature >38.3°C not due to another cause and/or leukocyte count <4000/mm^3^ or >12000/mm^3^) and with one or more respiratory signs (new onset of purulent sputum or change in character of sputum and/or worsening gas exchange plus at least one positive microbiological sample [quantitative cultures of a distal blind protected-specimen brush, with a threshold of 10^3^ colony-forming units/mL; or of a bronchoalveolar-lavage specimen, with a threshold of 10^4^ colony-forming units/mL; or of an endotracheal aspirate, with a threshold of 10^6^ colony-forming units/mL; or positive culture of a pleural-fluid specimen).

**Statistical analysis: Fine-and-Gray models**

Patients who developed CAPA had a minimum duration of mechanical ventilation (MV) or ICU length of stay (ICU LOS) before diagnosis, leading to a systematic classification bias when comparing with patients without CAPA. To limit this bias, we performed a Fine-and-Gray model considering CAPA as a time-dependent variable with death as the competing event.

*Mechanical ventilation*

For CAPA patients, 2 periods were created: a first period going from intubation to the diagnosis of aspergillosis and a second going from the diagnosis of aspergillosis to extubation.

8 patients were excluded of the model: 7 patients were still mechanically ventilated at day-90; 1 patient was diagnosed with CAPA 7 days after withdrawal of MV.

*ICU length of stay*

For CAPA patients, 2 periods were created: a first period going from ICU admission to the diagnosis of aspergillosis and a second going from the diagnosis of aspergillosis to ICU exit.

ICU length of stay was calculated as follow: time between ICU admission and exit if exit occurred before day-90; or time between ICU admission and death if death occurred before day-90; or 90 days if patient was still hospitalized in ICU at day-90.

**Table S1. Distribution of included patients according to participating ICU, mycological test available and CAPA status.**

|  | Patients included, n | CAPA incidence, n (%) |
| --- | --- | --- |
| Angers ^1^ | 111 | 0 (0%) |
| Brest ^2^ | 61 | 1 (1.6%) |
| Cholet ^3^ | 31 | 0 (0%) |
| Le Mans ^4^ | 85 | 4 (4.7%) |
| Lorient ^5^ | 28 | 0 (0%) |
| LRSY ^6^ | 48 | 0 (0%) |
| Morlaix ^7^ | 13 | 0 (0%) |
| * MIRNantes ^8^ | 97 | 3 (3.1%) |
| USIPNantes^9^ | 10 | 0 (0%) |
| Quimper ^10^ | 33 | 1 (3.2%) |
| RéaChir Rennes^11^ | 16 | 0 (0%) |
| * MIRRennes^12^ | 70 | 6 (8.6%) |
| SaintMalo^13^ | 32 | 2 (6.3%) |
| SaintNazaire^14^ | 32 | 1 (3.1%) |
| Vannes^15^ | 41 | 0 (0%) |

* Centers where patients were screened routinely for pulmonary aspergillosis

**Centers**

1: Medical Intensive Care, Angers University Hospital; 2: Medical Intensive Care, Brest University Hospital; 3: Division of Intensive Care, Cholet General Hospital; 4: Division of Intensive Care, Le Mans General Hospital; 5: Division of Intensive Care, Bretagne Sud General Hospital; 6: Division of Intensive Care, La Roche sur Yon General Hospital; 7: Division of Intensive Care, Morlaix General Hospital; 8: Medical Intensive Care, Nantes University Hospital; 9: Pulmonary intensive care unit, Nantes University Hospital; 10: Division of Intensive Care, Cornouille General Hospital; 11: Surgical Intensive Care, Rennes University Hospital; 12: Medical Intensive Care, Rennes University Hospital; 13: Division of Intensive Care, Saint Malo General Hospital; 14: Division of Intensive Care, Saint Nazaire General Hospital; 15: Division of Intensive Care, Vannes General Hospital

**Table S2: ECMM/ISHAM consensus criteria**

|  | Clinical | Radiological | Mycological |
| --- | --- | --- | --- |
| Entry criterion: ICU admission for COVID-19 | | | |
| Proven | - | - | Lung biopsy: Histopathologic or direct microscopic examination (hyphae + tissue damage) or positive culture/PCR from tissue |
| Probable CAPA (pulmonary form) | Refractory fever, pleural rub, chest pain, hemoptysis | Chest X-ray or CT scan:  • Pulmonary infiltrate  • Cavitating infiltrate | At least 1:  • Positive direct microscopy of BAL (hyphae)  • Positive culture of BAL  • Serum GM >0.5 or BAL GM ≥1  • Positive *Aspergillus* PCR in serum x2 or BAL x1 (<36 cycles) or serum + BAL x1 |
| Probable CAPA (tracheobron-chial form) | Bronchoscopic examination: airway ulceration, nodule, pseudomembrane, plaque or eschar | - | At least 1:  • Positive direct microscopy of BAL (hyphae)  • Positive culture of BAL  • Serum GM >0.5 or BAL GM ≥1  • Positive *Aspergillus* PCR in BAL x1 |
| Possible CAPA | Same as probable CAPA | Same as probable CAPA | At least 1:  • Positive direct microscopy of NBL (hyphae)  • Positive culture of NBL  • NBL GM >4.5 x1 or >1.2 x2  • NBL GM >1.2 + positive  • *Aspergillus* PCR in NBL |

ICU: intensive care unit; CAPA: COVID-19-associated pulmonary aspergillosis; CT: computed tomography; BAL: bronchoalveolar lavage; GM: galactomannan optical density index; PCR: polymerase chain reaction; NBL: nonbronchoscopic lavage

**Additional information about possible CAPA: baseline characteristics and outcomes**

**Table S3**. Baseline features at ICU admission of patients who met 2020 ECMM/ISHAM consensus criteria for possible CAPA compared to patients who met no criteria for CAPA

|  | **Possible CAPA**  **(n=17)** | **No CAPA**  **(n=67**3**)** | ***P* value** |
| --- | --- | --- | --- |
| ***Demographics, mean±SD or n (%)*** | | | |
| Age  (n=690) | 68.60±8.62 | 69.72±6.65 | 0.12 |
| Male  (n=690) | 14 (82.2) | 507 (73.48) | 0.58 |
| BMI (kg/m^²^)  (n=690) | 28.04±5.08 | 29.67±5.75 | 0.40 |
| ***Severity scores, mean±SD*** | | | |
| SAPS II  (n=642) | 39.94±13.93 | 38.56±12.93 | 0.79 |
| SOFA score  (n=626) | 4.88±2.61 | 5.20±2.83 | 0.37 |
| ***Comorbidities, mean±SD or n (%)*** | | | |
| Charlson Comorbidity Index  (n=690) | 3.94±2.28 | 3.80±2.53 | 0.76 |
| Diabetes  (n=690) | 4 (11.76) | 219 (31.74) | 0.60 |
| Hypertension (n=690) | 10 (58.8) | 377 (54.64) | 1 |
| Chronic respiratory failure  (n=690) | 3 (17.60) | 133 (19.28) | 1 |
| Chronic heart failure  (n=690) | 1 (5.90) | 108 (15.65) | 1 |
| Chronic kidney failure (n=690) | 1 (5.90) | 67 (9.71) | 1 |
| Cirrhosis (n=690) | 1 (5.90) | 38 (5.51) | 1 |
| Any immunosuppression  (n=690) | 3 (17.60) | 109 (15.80) | 0.74 |
| ***Treatments received for COVID-19, n (%)*** | | | |
| Corticosteroids  (n=690) | 10 (58.80) | 434 (62.99) | 0.81 |
| IL-6 antagonist  (n=690) | 0 (0) | 4 (0.59) | 1 |
| Hydroxychloroquine  (n=690) | 5 (29.40) | 90 (13.04) | 0.035 |
| Lopinavir/Rotinavir  (n=690) | 2 (11.70) | 84 (12.17) | 1 |
| Remdesivir  (n=690) | 1 (5.90) | 26 (3.77) | 0.48 |

CAPA: COVID-19-associated pulmonary aspergillosis; BMI: Body Mass Index; SAPS II: Simplified Acute Physiology Score version II; SOFA: Sequential Organ Failure Assessment

**Table S4**. Possible CAPA defined by 2020 ECMM/ISHAM consensus criteria: association with outcomes.

|  | No CAPA  (673) | Possible CAPA  (17) | *P* value |
| --- | --- | --- | --- |
| MV duration, days, mean±SD | 20.55±19.16 | 22.24±18.08 | 0.39 |
| ICU length of stay by day 90, days, mean±SD | 24.38±17.45 | 32.58±24.16 | 0.22 |
| Day-90 mortality, n (%) | 192 (27.83) | 6 (35.00) | 0.58 |

MV: endotracheal mechanical ventilation; ICU: intensive care unit

**Table S5.** Additional information about antifungal treatment in CAPA patients

CAPA: COVID-19-associated pulmonary aspergillosis; IQR: Interquartile range.

|  | **18 CAPA patients** |
| --- | --- |
| **Initiation of antifungal treatment, n(%)** | 17 (94) |
| **Time from CAPA diagnosis to first treatment (days), median [IQR]** | 1 [1-5] |
| **First antifungal treatment, n(%)** |  |
| Voriconazole | 15/17 (88) |
| Isavuconazole | 1/17 (6) |
| Caspofungin | 1/17 (6) |
| Liposomal Amphotericin B | 0/17 (0) |
| **Number of treatment lines used, n(%)** |  |
| 1 | 12/17 (71) |
| 2 | 5/17 (29) |
| 3 | 0/17 (0) |
| **Duration of antifungal treatment*, median [IQR]** | 19.5 [12.5-30.5] |

* Treatment duration was recorded even after ICU withdrawal, if applicable.

**Association between CAPA and MV duration, ICU length of stay, day-90 mortality.**

**Results of multivariate models.**

**Table S6.** Duration of mechanical ventilation.

|  | **HR (95% CI)** | ***P* value** |
| --- | --- | --- |
| CAPA | 0.66 (0.30-1.49) | 0.32 |
| Age (years) | 0.98 (0.97-0.99) | 0.001 |
| SOFA score at ICU admission | 0.91 (0.88-0.94) | p<0.001 |
| Immunocompromised status | 0.73 (0.55-0.98) | 0.03 |
| Charlson’s Comorbidity Index | 0.94 (0.89-0.98) | 0.008 |
| Time from symptom onset to ICU admission | 1.03 (1.00-1.05) | 0.03 |

CAPA: COVID-19-associated pulmonary aspergillosis; HR: hazards ratio; ICU: Intensive Care Unit; SOFA: Sequential Organ Failure Assessment; 95%CI: 95% confidence interval

**Table S7**. ICU length of stay.

|  | **HR (95% CI)** | ***P* value** |
| --- | --- | --- |
| CAPA | 0.61 (0.28-1.33) | 0.21 |
| Age (years) | 0.98 (0.97-0.99) | p<0.001 |
| SOFA score at ICU admission | 0.92 (0.88-0.95) | p<0.001 |
| Immunocompromised status | 0.72 (0.54-0.97) | 0.029 |
| Charlson’s Comorbidity Index | 0.92 (0.88-0.97) | 0.002 |
| Time from symptom onset to ICU admission | 1.03 (1.01-1.06) | 0.009 |

CAPA: COVID-19-associated pulmonary aspergillosis; HR: hazards ratio; ICU: Intensive Care Unit; SOFA: Sequential Organ Failure Assessment; 95%CI: 95% confidence interval

**Table S8.** Day-90 mortality.

|  | **HR (95% CI)** | ***P* value** |
| --- | --- | --- |
| CAPA | 2.07 (1.32-3.25) | 0.001 |
| Age (years) | 1.05 (1.03-1.08) | p<0.001 |
| SOFA score at ICU admission | 1.18 (1.12-1.24) | p<0.001 |
| Immunocompromised status | 1.68 (1.11-2.53) | 0.01 |
| Charlson’s Comorbidity Index | 1.03 (0.96-1.10) | 0.45 |
| Time from symptom onset to ICU admission | 1.01 (0.97-1.05) | 0.56 |

CAPA: COVID-19-associated pulmonary aspergillosis; HR: hazards ratio; ICU: Intensive Care Unit; SOFA: Sequential Organ Failure Assessment; 95%CI: 95% confidence interval
